# Antibacterial surface modification of titanium implants in orthopaedics

**DOI:** 10.1177/2041731418789838

**Published:** 2018-07-25

**Authors:** Wich Orapiriyakul, Peter S Young, Laila Damiati, Penelope M Tsimbouri

**Affiliations:** Centre for the Cellular Microenvironment, College of Medical, Veterinary & Life Sciences, Institute of Molecular, Cell and Systems Biology, University of Glasgow, Glasgow, UK

**Keywords:** Biomaterials, titanium, orthopaedic implants, topography, biofilms

## Abstract

The use of biomaterials in orthopaedics for joint replacement, fracture healing and bone regeneration is a rapidly expanding field. Infection of these biomaterials is a major healthcare burden, leading to significant morbidity and mortality. Furthermore, the cost to healthcare systems is increasing dramatically. With advances in implant design and production, research has predominately focussed on osseointegration; however, modification of implant material, surface topography and chemistry can also provide antibacterial activity. With the increasing burden of infection, it is vitally important that we consider the bacterial interaction with the biomaterial and the host when designing and manufacturing future implants. During this review, we will elucidate the interaction between patient, biomaterial surface and bacteria. We aim to review current and developing surface modifications with a view towards antibacterial orthopaedic implants for clinical applications.

## Introduction

Biomaterials are biological or synthetic substances that are designed to perform, enhance or replace the normal function of different tissues including skin, vasculature, bone, cartilage or tendon by interacting with the biological system. Ideal biomaterial properties vary depending on the tissue being replaced and required function. Ideal biomaterials are highly biocompatible, often with specific functionalisation usually serving as a matrix for cell adhesion that regulates cell processes such as proliferation, migration and matrix synthesis. The ideal biomaterial in orthopaedics would be highly biocompatible, inexpensive and straightforward to manufacture, reproducing the function of the replaced tissue such as stimulating the production of new bone. A great deal of research has been dedicated to developing ideal biomaterials for orthopaedics particularly focussed on osseointegration.

However, any surgical intervention, particularly with implantation of biomaterials, runs the risk of biomaterial-associated infection (BAI) or periprosthetic joint infections (PJI). Bacteria adhere to biomaterial surfaces, where they produce biofilms enhancing their colonisation, preventing phagocytosis and evading the immune response, as well as becoming more resistant to systemic antibiotics.^1–4^ BAI cause significant patient morbidity and mortality, contributing to implant failure and loosening with an average failure rate of 2%–5%.^5–7^ Patients often require extensive further surgical intervention and long-term antibiotic therapy. The average economic cost of each patient with PJI is £25,000.^8^ An ideal biomaterial would also provide appropriate antibacterial action.

Biomaterial function differs depending upon the indication, and subsequently the infecting organisms may vary. In trauma, for example, skin commensals such as *Staphylococcus aureus* (*S. aureu*s) and *Staphylococcus epidermidis* (*S. epidermidis*) are the most common pathogens, however, contamination from the environment at the time of injury can introduce a wide spectrum of bacteria to the fracture and soft tissues.^9^ Biomaterials used in fracture fixation are largely designed to promote fracture union without osseointegrating themselves. In elective orthopaedics, PJI affects approximately 1% of primary arthroplasties, often leading to poor outcome.^10,11^ In PJI, the most common bacteria are again *S. epidermidis* and *S. aureu*s, but other species including *methicillin-resistant Staphylococcus aureus (MRSA)* and Gram-negative organisms such as *Pseudomonas aeruginosa (P. aeruginosa)* may be seen.^12–15^ Due to the rise in rates of arthroplasty and trend towards younger patients with higher expectations, uncemented implants have gained popularity.^16^ These implants rely upon osseointegration with the host bone to create a secure long-term fixation at the bone–material interface, which must be maintained through continuous cycles of bone remodelling. In order to optimise implant osseointegration, research has been directed at developing implant surfaces that encourage new bone formation without significant consideration towards bacterial response.

Treatment of PJI requires a multimodal treatment strategy. Surgery is usually necessary to reduce the localised bacterial infection and remove the biofilm-coated implants, along with long-term systemic antibiotic drug administration.^17,18^ Local antibiotic treatment strategies such as polymethylmethacrylate (PMMA) antibiotic beads and high-dose antibiotic cement spacers show good clinical results;^19,20^ however, overdose of local and systemic antibiotics might negatively affect osteogenesis and are not without complications.^21,22^ Use of surface-modified orthopaedic implants is another option, which may provide clinical advantage. For example, reducing the initial risk of bacterial infection from early or late haematogenous spread, reducing local and systemic antibiotic toxicity effects and improving clearance of infection and subsequent osseointegration following established infection.

Understanding the interaction between host, biomaterial and microorganism is very important for the development of antibacterial orthopaedic implants for clinical applications. This review highlights the relationship between host cells, implant materials and bacteria from an osteoimmunological aspect. We also focus on current surface fabrication techniques of Ti and its alloys for the development of antibacterial surface modifications and their potential clinical applications.

## Interaction between host, material and bacteria

**Figure 1.**
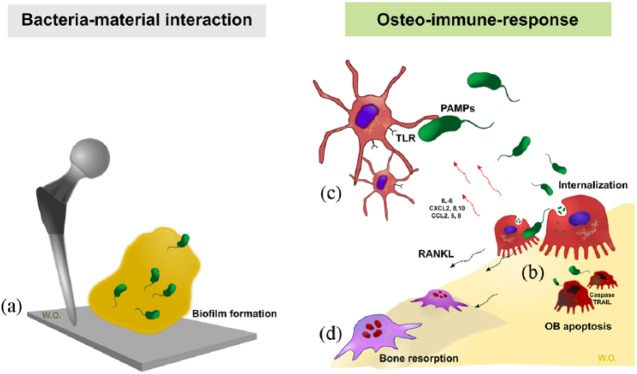
Bacteria–material–host interaction. (a) Bacteria adhere on material surface and form a biofilm enhancing their proliferation and protecting themselves from immune response and antibiotic drugs. (b) Bacteria interact with host cells such as osteoblasts. Osteoblasts non-professionally internalise bacteria.^23^ This mechanism helps bacteria evade the immune system.^24^ Bacteria induce osteoblast apoptosis by toxin production.^25,26^ Infected osteoblasts also induce tumour necrosis factor–related apoptosis-induced ligand (TRAIL) via caspase-8.^27^ (c) Immune cells, both innate and adaptive, attack the planktonic bacteria to reduce bacterial numbers. Infected osteoblasts produce cytokines to activate immune response. (d) Infected osteoblasts produce RANKL, CXCL2 and CCL3 which enhance osteoclastogenesis resulting in bone resorption.^28^,^29^ OB: osteoblast; PAMPs: pathogen-associated molecular patterns; TLR: toll-like receptors.

The host reacts to microorganisms via innate and acquired immune responses, though some bacteria can evade this by producing a biofilm or by becoming internalised into the osteoblast cells (Figure 1). Planktonic state bacteria are initially attracted to a material surface by different forces, for example, van der Waals or gravitational forces. Once the bacteria have adhered to the surface, they form stronger adhesion using pili. These bacteria then form colonies on the implant surface and secrete extracellular matrix rich in polysaccharides and proteins to form a biofilm layer, hence protecting themselves from the immune system. Bacteria and biofilm formation can reduce osteoblast viability and disturb osteoblast–osteoclast interaction resulting in bone resorption.^13,28^ A better understanding of the host cellular and bacteria–material surface interactions will help to improve treatment of PJI as well as improve material design. The ideal biomaterial will reduce bacterial colony formation (by anti-adhesive or bactericidal effect), promote osteogenic induction and maintain long-term implant osseointegration.

### Osteoimmunology: how bone cells respond to bacteria

Once bacterial pathogens are introduced into the host via direct surgical site or haematogenous spread, bacteria then present pathogen-associated molecular patterns (PAMPs) such as lipopolysaccharide (LPS), lipoprotein, lipoteichoic acid (LTA) or peptidoglycans.^30–32^ Bacterial detection results in activation of the complement cascade and attracts inflammatory cells to the infected site. Phagocytic innate immune cells, such as macrophage and neutrophils engulf and kill planktonic bacteria directly. They can recognise bacterial PAMPs via toll-like receptors (TLR), a family of cell surface pattern recognition receptors (PRRs). Ligation of TLRs activates intracellular nuclear factor kappa B (NFκB) signalling cascades, which results in the increased production and release of soluble chemoattractants (cytokines and chemokines). These recruit immune cells to the site of infection.^32^ Professional antigen presenting cells such as dendritic cells (DCs) link the host innate and adaptive immune responses, and by activating both cytotoxic CD8^+^T-lymphocytes and B lymphocytes produce antibodies against bacteria.^33^ Dysregulation of this process can result in an attenuated immune response, driving sustained chronic infection.

Osteoblasts respond to planktonic bacteria by several mechanisms.^34^ Initially, osteoblasts can internalise bacteria into vesicles; however, some bacteria have adapted to remain quiescent or to secrete toxins such as phenol-soluble modulins (PSMs) to escape internalisation and induce osteoblast necrosis and apoptosis. These bacteria will then continue to infect surrounding cells. Infected osteoblasts also activate innate and adaptive immune cells by producing a plethora of cytokines and chemokines (such as interleukin-6 (IL-6), CXCL2, CXCL8, CXCL10, CCL2, CCL3 and CCL5).^33,35^ Infected osteoblasts also produce factors such as RANKL, granulocyte macrophage colony stimulating factor (GM-CSF), macrophage colony stimulating factor (M-CSF) and granulocyte colony stimulating factor (G-CSF) to enhance osteoclastogenesis leading to bone resorption.^33,36^

Osteoclasts originate from haematopoietic stem cells and differentiate from the same precursors as macrophages and dendritic cells.^34^ Li et al.^37^ demonstrated that mature osteoclasts can function as antigen-presenting cells and can activate CD4 ^+^ and CD8 ^+^ T cells. Osteoclast precursors are attracted to sites of infection by sphingosine-1-phosphate (S1P).^38^ During the host response to a bacterial infection, macrophages become activated by the inflammatory environment and produce pro-inflammatory cytokines, which further promote osteoclastogenesis.^39^

Understanding the immunological response to common microorganisms is necessary for treatment of osteomyelitis and PJI. *S. aureus*, for example, binds to bone extracellular matrix using adhesion proteins termed microbial surface components recognising adhesive matrix molecules (MSCRAMMs). These include collagen-binding adhesin (Cna, binding to collagen), fibronectin-binding protein (FnBP; binding to fibronectin),^40,41^ bone sialoprotein binding protein (Bbp; binding to bone sialoprotein) and major histocompatibility complex class II (MHC class II; binding to osteopontin).^42^

FnbA and B as well as Cna play important roles in bacterial binding to implants.^43^ Testoni et al.^44^ suggested Cna and Bbp synergised to drive *S. aureus*-osteoblast adhesion. Bacterial fibronectin-binding proteins (FnBPs) binding to osteoblast α_1_β_5_ integrins trigger the non-professional phagocytic process which internalises bacteria.^45^ After *S. aureus* binds to the bone extracellular matrix, osteoblasts engulf *S. aureus cells*, a process dependent upon cytoskeletal proteins, such as actin.^23^ This then supports *S. aureus* evading the immune system and promotes bacterial spreading.

### Bacterial–material interaction: underlying theories, interaction phases and biofilm formation

**Figure 2.**
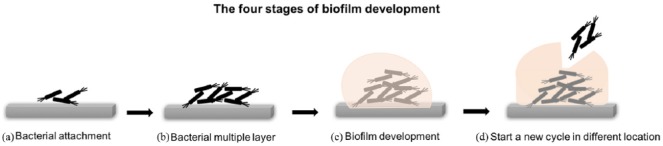
The four stages of biofilm development. (a) Initial bacterial attachment. (b) Bacteria start to produce multiple layers through cell aggregation and accumulation. (c) Biofilm development and matrix elaboration. (d) Bacteria start a new cycle of biofilm formation in different location.

There are three main influences on bacterial interaction with material surfaces, material features, bacterial features and the environment (Figure 2).

### Bacterial features

Different bacterial species have different adherent behaviour to biomaterial surfaces due to their physicochemical characteristics and preferred environment.^46,47^

#### Bacterial hydrophobicity/hydrophilicity

In general, bacterial species with hydrophobic properties prefer binding to hydrophobic surfaces and vice versa. However, the material surface hydrophobicity plays a more important role in bacterial adhesion than the intrinsic bacterial surface hydrophobicity.^46^ Bacterial hydrophobicity changes according to bacterial age, surface structure and medium growth. Possible reasons behind this include increased exopolysaccharide production with higher salt concentrations and in aged cells and lower nutrients in the culture media. These factors all lead to a drop in hydrophobicity.^48^

#### Bacterial surface charge

The relationship between bacterial surface charge properties and bacterial adhesion are not clearly understood. However, there is some evidence that bacterial charge is affected by growth medium, environmental pH, the buffer ionic strength, bacterial age and surface structure. Moreover, bacteria in aqueous solutions are usually negatively charged.^46,47^ Hence, the surface charge for both bacterial and biomaterial should be considered in predicting bacterial adhesion on material surfaces.^49^

There are two phases of the bacterial–material interaction. Phase I: this is the initial, instantaneous and reversible physical phase. Phase II: this is the time-dependent and irreversible molecular and cellular phase.^47^ Biomaterial surface topography such as pattern and roughness can affect the bacterial adhesion.^50^

Normally, bacteria prefer to grow on available surfaces rather than in the surrounding aqueous phase.^51^ During phase I, bacterial adhesion starts with surface attraction, followed by cell adsorption and attachment.^52^ The bacterial movement occurs by physical interactions such as Brownian motion, van der Waals and gravitational forces; the surface electrostatic charge and hydrophobic interactions.^25,29^ In addition, physical interactions are classified into long- and short-range interactions. In long-range interactions, the distance between cells and surfaces is not specific (>50 nm). While in short-range interactions the cells have a close contact (< 5 nm) with the surface. Once the bacteria have attached to the surface (long-range interactions), the initial part of adhesion occurs (short-range interactions), allowing phase II to begin.^47,53^

There are three theories described to date that determine the interaction between bacterial cells and surfaces. First, the Derjaguin–Landau–Verwey–Overbeek (DLVO) theory describes the net interaction between cells and surfaces when the particle adhesion is affected by long-range interactions. These include Lifshitz-van der Waals interactions and overlapping electric double-layer interactions.^54^ This explains the reason why some colloidal systems agglomerate while others do not. Second, the thermodynamic theory^55^ describes bacterial attachment to the surfaces with different attractive and repulsive interactions like van der Waals, electrostatic or dipole. Generally, the thermodynamic theory works as a closed system where the organism converts the substrate to energy without any energy from the outside.^56^ Finally, a combination of the available theories termed the extended DLVO theory was developed. This includes the hydrophobic/ hydrophilic interactions.^57,58^

In the second phase (adhesion phase), molecular-specific reactions occur between structures on bacterial surfaces with the substratum surfaces. Specific polymeric structures in bacteria such as capsules, fimbriae or pili serve as bridges between the cell and the surface.^53,59,60^ Clumping factors, proteins and teichoic acid are factors that may play a role in highly viscous masses.^46^ Following phase II, bacteria may then begin to form biofilms on the material surface.

### Biofilm formation on implants

**Figure 3.**
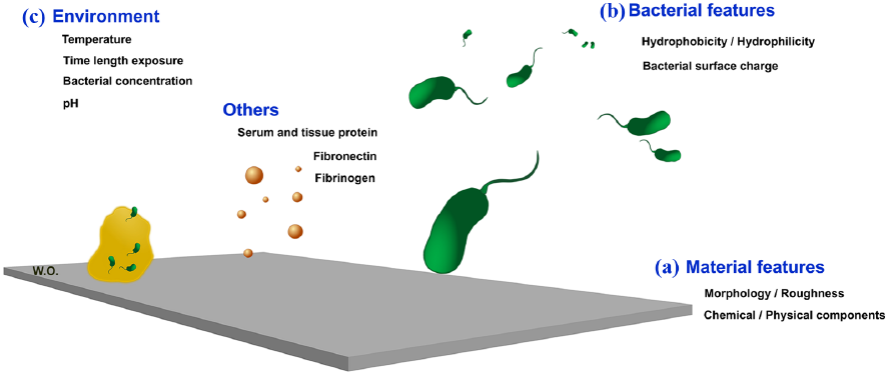
Three main features affect bacterial–material interaction. (a) Material features such as morphology and physicochemical cues. (b) Bacterial features including surface charge and hydrophobicity/hydrophilicity. (c) Environments such as temperature, pH, bacterial concentration and contact time as well as other factors such as serum and protein.

Biofilm formation is an advantageous process for bacteria. The majority of the world’s bacterial populations are found in the form of a biofilm at various stages of development.^61^ A biofilm is a structured group of bacteria that cover themselves in an exopolysaccharide matrix, which results in firm adhesion on the implant. Inside biofilms, intercellular bacterial communication regulates gene expression and adaptation including phenotypic variation and survival during nutrient starvation.^62,63^ In addition, the bacteria are protected from antibiotics and dynamic environments.^64,65^ There are four steps for biofilm formation (Figure 3). Step 1: The bacteria initially attach on the substrate. Step 2: Accumulation of multiple cell layers through cell aggregation and accumulation. Step 3: matrix elaboration and biofilm development. Step 4: Bacterial release to start a new cycle of biofilm formation in a proximal location.^43,66,67^ Following biofilm development, bacteria work in groups rather than as individual cells in a process called ‘quorum sensing’. A number of methods have been developed to prevent biofilm formation, including inhibition of quorum sensing, anti-adhesion drugs and macromolecules.^43,68–72^ However, we will focus on material surface adaptations.

### Material features

There are many factors influencing bacteria adherence to biomaterials surfaces including surface morphology and roughness, surface chemical composition and surface hydrophobicity or hydrophilicity.

#### Surface morphology and roughness

It has become clear that the micro/nano-topography of a biomaterial plays an important role in bacterial adhesion.^73–79^ This concept originates from observations made on the unique features of Cicada (*Psaltoda claripennis*) wings, which have nanoscale pillar patterns protecting the insect from bacterial infection.^80^ Bacteria prefer rough or grooved surfaces that increase the contact area and enhance the binding ability when compared to flat surfaces.^81,82^ Moreover, use of polymer coatings on material surfaces reduces the biofilm deposition and bacterial adhesion.^82^ The coating reduces the size of micro- or nano-grooves in the material, which become too small for the bacterium to fit, thus shrinking the potential contact area between the bacterium and binding sites.^83^

Various studies have published the effects of topographical modifications on bacterial adhesion and survival. Tsimbouri et al.^84^ showed that TiO_2_ nanowires produced by hydrothermal oxidation reduce the *P. aeruginosa* growth in the early stage of bacterial adhesion compared with polished Titanium (Ti). Truong et al.^85^ reported that eukaryotic and prokaryotic cell attachment on Ti surfaces can be controlled by modifying the surface topography and morphology into micro- or nano-structures. Teughels et al.^86^ reported that high surface roughness assisted biofilm formation on implants. Pier-Francesco et al.^87^ found that the *P. gingivalis* adhesion to titanium was inhibited at surface roughness levels below *R_a_* 350 nm, a roughness level generally encountered for implant collars/abutments. Several authors have demonstrated that TiO_2_ photo-activation leads to loss of viability for five different pathogens (*Escherichia coli* (*E. coli*), *P. aeruginosa, S. aureus, Enterococcus hirae (E. hirae)* and *Bacteroides fragilis (B. fragilis)*).^88–92^ According to Verdier et al.,^93^ TiO_2_ under ultraviolet (UV) irradiation showed an antibacterial activity for the *E. coli*.

#### Surface chemical/physical composition

Surface chemistry can play a role in bacterial adhesion and proliferation. Materials with different functional groups change bacterial adhesion depending on material hydrophobicity/hydrophilicity and charge state.^46,47^ Water contact angle (WCA) measurements have been used to reveal the hydrophobic (high) or hydrophilic (low) nature of material surfaces. Metals tend to have a high surface energy, negative charge and hydrophilic features as confirmed by WCA, whereas polymers have low surface energy, less charge and hydrophobic features.^47^ In addition, the hydrophilicity of materials may change with time, for example, Ti(OH)_4_ hydrophilicity may decline overtime due to air oxidation or carbon contamination and become TiO_2_ (hydrophobic).^94–96^ Thus, it is important to monitor the chemical changes in the surface features over time to determine the bacteria survival time.^97,98^

### The environment

General environment factors such as temperature, time length exposure, chemical treatment, pH, antibiotic presence and bacterial concentration may play a role in bacterial adhesion.^46,47,99^ Optimum bacterial temperature allows bacterial enzyme activity, bacterial growth and biofilm formation.^99,100^ Temperature changes may also affect the physical properties, for example, at 35°C bacteria have a single flagellum, while at 21°C they have 2–3 flagella and at 10°C they have multiple flagella. At lower temperatures, the ability of biofilm adhesion increases if properties of polysaccharides are uniform.^101^ This suggests that by raising the temperature, adhesion will reduce between the bacteria and the substrate. However, despite increasing temperature to 80°C–90°C, biofilm removal is not improved due to ‘baking effects’.^102^

Changing pH and environmental chemical concentrations such as NaCl, HCl and KCl affect bacterial growth.^99,103–105^ Bacteria have the ability to respond to changes in internal or external pH by adjusting their activity and protein synthesis.^106^ This allows the bacteria to adapt to small changes in their environment.^99,107^ Some bacteria have the ability to modify their metabolism to lower growth rate under specific drug pressures until they find favourable conditions for multiplication.^108^ This can be favourable for bacteria as some antibiotics act to decrease bacterial adhesion.^109,110^

### Other factors: serum or tissue proteins

Serum or tissue proteins such as fibronectin (FN), fibrinogen (Fg) and albumin may promote or inhibit bacterial biofilm accumulation on biomaterial surfaces.^46,47^

*Fibronectin* is an extracellular glycoprotein that is found in soluble and insoluble forms in extracellular fluids and connective tissues.^111–113^ FN plays various roles in cellular activities such as adhesive interactions between cell surface integrin receptors by organising into a fibrillar network;^114^ development;^115^ wound healing; haemostasis and tissue repair.^116^ Moreover, interactions between growth factors and FN control growth factor presentation and their activity.^117^

Fibrinogen (Fg) is a protein that plays a role in blood coagulation, platelet adhesion and aggregation and hemostatic processes.^118,119^

## Titanium surface material modification

A wide variety of different materials are used as biomaterials; however, we will specifically focus on the surface modification of titanium in this review. Ti and its alloys (Ti-6Al-4V, Ti-5Al-2·5Fe and Ti-6Al-7Nb) are one of the most widely used materials in orthopaedics, both in trauma and elective practice. Ti has good corrosion resistance, high strength, low weight and modulus of elasticity much closer to that of bone than other metals. The benefit of using Ti-based alloys is their non-reactivity due to auto-passivation. However, the bioinert nature of Ti also means that as a flat surface, it shows no osteoinduction. Titanium is a very adaptable material, and many techniques have been used to modify surface roughness and create interconnecting porous architecture in order to promote osseointegration. Common microscale surface modification techniques for improved osseointegration can be divided into surface roughening (such as blasting, plasma spray, meshing, etching and anodisation) and surface coating (such as plasma-sprayed hydroxyapatite (HA) coating). Currently marketed implants include porous coating (e.g. Zimmer, CSTi); plasma-sprayed HA coating (e.g. DePuy Synthes, PureFix; Stryker); HA coated on porous plasma spray titanium alloy (BoneMaster; Biomet) and Sintered–titanium bead with plasma-sprayed HA coat (ROUGHCOAT; Smith & Nephew). Clinical trials have reported good clinical outcomes and prosthetic longevity with cementless fixation.^50,120^ However, the effect of macro- to microscale surface roughness on bone ingrowth remains inconclusive.^121,122^ Many of these techniques create bone on-growth rather than ingrowth and suffer from weak bonding between the surface layer and the underlying implant, with shearing and failure of the surface.^123^ Furthermore, the effect of these surface modifications on bacterial adhesion and biofilm formation has been poorly studied.

The design of prostheses with surfaces that enhance osseointegration and osteoinduction, while also giving an antibacterial effect without cytotoxicity would be ideal, though it remains challenging. Nanotopographical surface modification is an interesting candidate for orthopaedic implants. The current success of nanoscale surface feature design is due not only to promotion of osteogenesis but some surface features also prevent bacterial adhesion. Therefore, understanding the difference between surface patterns and their interaction with osteoblasts and bacteria is crucial for nanotopographic design. Puckett et al. studied bacterial adhesion on different nanotopographic patterns on titanium including nanotubular, nanotextured and nanorough. Nanorough created by electron beam evaporation decreased adhesion of *S. aureus, S. epidermidis* and *P. Aeruginosa*, while nanotubular and nanorough fabricated by anodisation increased bacterial adhesion.^124^

### Topographical modification for improved osteointegration

There are various techniques for patterning material surfaces at the nanoscale such as photolithography, polymer demixing, electron beam lithography and anodisation, the more common examples are summarised in Table 1.^136^ Many of these show induction of osteogenesis including nanotubes,^126^ nanopits,^137^ nanopores^138^ and nanopillars.^132^

**Table 1.** Examples of nanopatterning on titanium surface and fabrication techniques.

Nanotopography	Materials	Technique	Reference
Nanotubes	Titania	Template-assisted method	Tan et al.^125^
		Anodisation	Gulati et al.,^126^ Park et al.,^127^ Pozio et al.^128^ and Oh et al.^129^
		Hydrothermal	Liu et al.^130^
Nanowires	Titanium	Hydrothermal	Tsimbouri et al.^84^ and Pan et al.^131^
Nanotexture	Titanium	Anodisation	Puckett et al.^124^
Nanopillars	Titanium	Anodisation	Sjöström and colleagues^132,133^
Nanophase	Titania	Sintering	Webster et al.^134^
Nanorod	Titanium	Anodisation	Ning et al.^135^

## Surface antibacterial modification

Ideal antibacterial implant coatings should be biocompatible with no local or systemic toxicity, easy to use with proven antibacterial effects, as well as inexpensive and easy to manufacture.^139^ The currently available products in the market are shown in Table 2. The most commonly used involve either antibiotic loading or silver ions; however, these products are expensive, cause local cell toxicity and the long-term side effects and clinical outcomes require further study.

**Table 2.** Examples of available antibacterial techniques and orthopaedic implants in the market.

Products	Brand	Technique	Outcomes	References
Antibiotic-coated tibial nail	PROtect, Synthes	Titanium alloy tibial nail coated with gentamicin sulphate	19 patients, good fracture healing	Fuchs et al.^140^
Antibiotic-coated external fixator pins	OrthoGuard AB, Smith & Nephew	Gentamicin-coated polyurethane sleeve	In vitro, >80 µg/mL at 2 h and 1 day elution time points, >4 µg/mL MIC breakpoint for at least 4 weeks	Forster et al.^141^
Antibiotic-loaded hydrogel for implant coating	Defensive antibacterial coating, DAC, Novagenit, Italy	Antibiotic-loaded degradable hydrogel-linked hyaluronan and poly(d,l-lactide)	Reduce rate of post-surgical site infections after internal fixation in closed fractures	Drago et al.^142^ and Malizos et al.^143^
Silver ions–coated titanium alloy endoprosthesis	Agluna, Accentus	Anodisation of titanium implant	*N* = 170, lower infection rate compared to control	Wafa et al.^144^

MIC: minimum inhibitory concentration.

**Figure 4.**
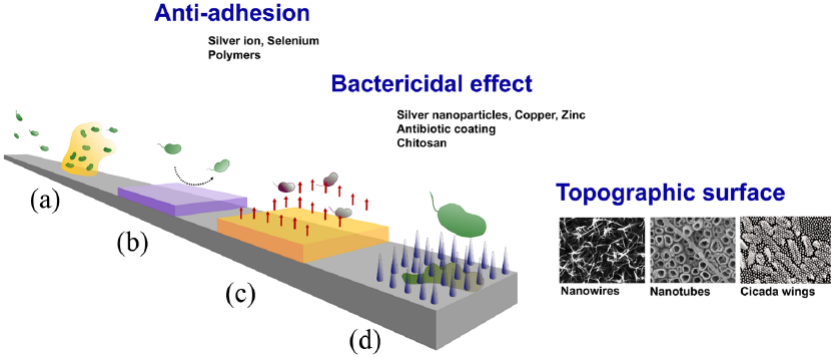
Planktonic bacteria attach on material surface and form biofilms. (a) Various techniques were used as antibacterial strategies. Anti-adhesive surface coats using concepts of surface chemistry and functionality including ions^145^ and polymer coats.^146^ (b) Material surface can be coated with bactericidal substances such as antibiotics^147^ and silver.^148^ (c) Nanotopographic surface modifications were also effective strategies used as either anti-adhesives or bactericidal. (d) The examples of nanotopography, such as nanowires promoting osteoblastogenesis and have bactericidal effects.^84^ Other bactericidal topographies include nanotubes (permission from Yu et al.^149^) and cicada wings (permission from Ivanova et al.^80^).

Current techniques for reducing bacterial attachment and biofilm formation include anti-adhesive function and bactericidal function. Examples of available strategies for antibacterial treatment including surface coating, nanotopography, as well as dual-function are shown in Figure 4 and Tables 3 and 4.

**Table 3.** Examples of surface coating.

	Coating substrate	Coating technique	Material	Bacteria	References
Bactericidal	Silver	Galvanic deposition	Titanium	*Staphylococcus aureus*	Gosheger et al.^148^
	Zinc oxide nanoparticles	EHDA deposition	Glass	*S. aureus*	Memarzadeh et al.^150^
	Iodine	Anodic oxidation coating	Titanium pins	*S. aureus* Escherichia coli	Shirai and colleagues^151,152^
	Chitosan–vancomycin	Electrophoretic deposition	Titanium	*S. aureus*	Ordikhani et al.^153^
	Silver and copper ion implantation	Ion implantation with MEVVA ion source	317L stainless steel Titanium, titanium alloy	*S. aureus*	Wan et al.^154^
Anti-adhesion	Silicon ions	Ion implanter with Si sputtering targets	316LVM stainless steel	*S. aureus* Staphylococcus epidermidis	Braceras et al.^145^
	Selenium	Dried in laminar airflow conditions	Titanium alloy	*S. aureus* S. epidermidis	Holinka et al.^155^
	Poly(ethylene glycol)-based polymer coating	Spin-coating	Glass	*S. aureus* S. epidermidis	Saldarriaga Fernández et al.^146^
	Polyethylene oxide	Directly exposed	Silicon rubber sheet	*S. aureus* S. epidermidis	Nejadnik et al.^156^
Dual function (anti-bacteria and promote osteogenesis)	Poly(l-lysine)-grafted-poly(ethylene glycol) and RGD	Direct adsorption	Titanium oxide	*S. aureus*	Harris et al.^157^
	Dextran-BMP2	Dopamine	Ti-6Al-4V	*S. aureus* S. epidermidis	Shi et al.^158^
	Surface-grafted Chitosan and RGD peptide	Dopamine-glutaraldehyde anchoring	Ti-6Al-4V	*S. aureus* S. epidermidis	Shi et al.^159^

EHDA: electrohydrodynamic atomisation; MEVVA: metal vapor vacuum arc.

**Table 4.** Examples of topographic surface modification.

	Topographic patterns	Fabrication techniques	Materials	Bacteria	References
Bactericidal	Gecko-skin	–	*Luciobarbus steindachneri*	*Porphyromonas gingivalis*	Watson et al.^160^
	Nanopillars (Cicada-inspired)	–	*Psaltoda claripennis*	*Pseudomonas aeruginosa*	Ivanova et al.^80^
	Nanopillars	Reactive-ion beam etching	Black silicon	*P. aeruginosa*	Ivanova et al.^161^
	Nanowire array (brush type/niche type) (Cicada-inspired)	Alkaline hydrothermal	TiO_2_	*P. aeruginosa*	Diu et al.^162^
	Micro-nano (dragonfly wings inspired)	Hydrothermal etching	Titanium	*P. aeruginosa*	Bhadra et al.^163^
Anti-adhesion	Lotus leaf-inspired (*Nelumbo nucifera*)	Femtosecond laser ablation	Titanium	*Staphylococcus aureus* Staphylococcus epidermidis	Truong et al.^164^
	Lamella-like	Direct laser interference patterning (DLIP)	Polystyrene	*S. aureus*	Valle et al.^165^
	Microscale cross patterned	Moulding	Polydimethylsiloxane (PDMS) urinary catheter	*Enterobacter. cloacae*	Vasudevan et al.^166^
	Sharklet micropattern (shark skin-inspired)	Emboss/cast	Polydimethylsiloxane elastomer, acrylic films	*S. aureus (MSSA, MRSA)*	Mann et al.^167^
Dual function (anti-bacteria and promote osteogenesis)	Nano-microphase grain	Compacts and sintered	ZnO and TiO_2_	*S. epidermidis*	Colon et al.^89^
	Topography and chemical patterns	Pulsed plasma polymerisation and UV-irradiation	Silicon wafers	*E. coli*	Ploux et al.^168^
	Nanowires	Hydrothermal treatment	TiO_2_	*P. aeruginosa*	Tsimbouri et al.^84^
	Sr- and Ag-loaded nanotubes	Anodised titanium, Sr(OH)_2_ hydrothermal and soaked in AgNO_3_	Titanium foils	*S. aureus (MRSA and MSSA)* E. coli	Cheng et al.^169^
	Zn incorporated nanotubes	Anodisation and hydrothermal treatment in Zn-containing solutions	Titanium	*S. aureus*	Huo et al.^170^

MRSA: methicillin-resistant Staphylococcus aureus; MSSA: methicillin susceptible Staphylococcus aureus.

### Anti-adhesive function

#### Topographic modification

The effects of microscale topography on bacterial adhesion remain controversial. Whitehead et al.^90^ demonstrated that bacteria can be retained in pits (substratum layer) depending on bacterial size and pit size, however, other authors have suggested that microscale surface roughness does not affect bacterial adhesion.^171,172^ Interestingly, some nanoscale topographic features can promote cell differentiation while decreasing bacterial adhesion. Ploux et al.^168^ created nanoscale pattern on silicon wafers by UV-photolithography. This surface reduced bacterial adhesion and allowed human osteoprogenitor cell adhesion.

#### Surface chemistry modification/surface coating

##### Ionic implantation/element coating

Silver ion coating of biomaterials has been widely studied. Della Valle et al.^173^ used anodic spark deposition (ASD) treatment to incorporate chemical elements such as silver nanoparticles, calcium or silicon into titanium oxide. They showed that the silver nanoparticle coating reduced bacterial adhesion, as well as being biologically safe. Combinations of nanotopographic surfaces and silver coating have also been studied. Das et al.^174^ fabricated titania nanotubes by anodisation and subsequently treated with silver deposition. These nanotubes showed good osteoblast adhesion and proliferation, while *P. aeruginosa* colonies were reduced.

Selenium is another element that can inhibit bacterial attachment. Holinka et al.^155^ studied the effect of sodium selenite coating of titanium discs on bacterial adhesion and showed that it can reduce *S. epidermidis* adhesion without significant changes in the growth of MG63 cells.

##### Surface functionality (receptor/ligand interaction)

Polymer brush is a technique attaching polymer chains such as polyethylene glycol (PEG), polyethylene oxide (PEO)^156,175^ onto surfaces to prevent bacterial adhesion and protein adsorption. Nejadnik et al.^156^ showed that PEO can reduce *Staphylococci* adhesion; however, polymer brush coatings are easily removed by even fluid shear, which is challenging for clinical applications.

### Bactericidal function

#### Topography modification

Ivanova et al.^80^ first noted the antibacterial effect of cicada wings (*P. claripennis*) against *P. aeruginosa*. The nanoscale pillars seen on cicada and dragonfly wings exhibit not only a self-cleaning property, as shown by the reduction of WCA, but also incorporate bactericidal effects. Hasan et al.^176^ further showed that these wing nanopillars effectively clear Gram-negative bacteria. Ivanova et al.^161^ then fabricated nanosurface patterns on black silicon by reactive-ion beam etching. This technique created nanofeatures mimicking dragonfly wings (*Diplacodes bipunctata*), which also showed effective bactericidal properties. Further to these studies, several authors have reported the antibacterial efficacy of titania nanowires by hydrothermal oxidation of titanium surfaces. Diu et al.^162^ engineered hydrothermal-treated nanowire arrays that can damage the bacterial membrane while also promoting adhesion and proliferation of MG63 cells. Bhadra et al.^163^ showed not only the bactericidal effect of nanowire arrays on *P. aeruginosa* but also their ability to enhance fibroblast proliferation. Tsimbouri et al.^84^ elegantly elucidated the osteogenic effect of hydrothermally treated titania nanowires. Using an osteoblast–osteoclast co-culture, they showed that nanowires reduce osteoclast maturation, promote osteogenesis as well as confirm the bactericidal effect on *P. aeruginosa*.

#### Surface chemistry modification/surface coating

*Metals, for example, silver, zinc and copper*. Silver nanoparticles can inhibit bacterial growth and have low risk of development of bacterial resistance.^177^ Gosheger et al.^148^ studied the bactericidal effectiveness of silver-coated mega-endoprostheses in a rabbit model. They showed significantly lower infection rates when compared to the control group. Copper and zinc also show bactericidal effects by providing oxidative stress, protein dysfunction and membrane damage.^178^ Memarzadeh et al.^150^ showed that zinc oxide nanoparticle coating inhibited *S. aureus* adhesion and promoted osteoblast growth.

*Non-metal elements, for example, selenium and iodine*. Shirai et al.^151^ demonstrated that iodine-supported titanium can inhibit bacterial colonisation and promote osteoconductivity, noted by osteoid formation surrounding titanium external fixator pins. Tsuchiya et al.^152^ conducted a clinical study in 222 patients using iodine supports by anodic oxidation coating. The results revealed effective infection prevention without adverse effects.

##### Antimicrobial polymers

Timofeeva and Kleshcheva^179^ described the use of positively charged polymers (such as quaternary ammonium or phosphonium polymers) to attack bacterial surfaces, which are negatively charged. These polymers act as surfactants which can damage bacterial cell walls and cell membranes, resulting in cell lysis.^180^

#### *Organic origin*, for example, antibiotics, anti-infective peptides and chitosan

##### Antibiotic coating

There are many ways to deliver antibiotic drugs such as loading antibiotic in bone cement or degradable materials and superficial modification of materials through covalently binding antibiotics or composite materials consisting of antibiotics embedded in gel or solid matrix.^181^ Examples of antibiotic elution are gentamicin-loaded poly-l-lactide (PLLA) and gentamicin-loaded poly(d,l-lactide) (PDLLA).^168^ Kazemzadeh-Narbat et al.^182^ showed that the use of cationic antimicrobial peptides combined with HA coating on titanium worked against *P. aeruginosa* in vitro. Systemic side effects of antibiotic-loaded materials are rare; however, antibiotic resistance and bone ingrowth disturbance must be considered.

##### Chitosan coating

Chitosan itself has bactericidal effects^183^ and can be used as a drug-eluting coating. Ordikhani et al.^153^ coated titanium surfaces with chitosan–vancomycin composite by a cathodic electrophoretic deposition technique. Lin et al.^184^ and Yang et al. fabricated quaternized chitosan derivative (hydroxypropyltrimethyl ammonium chloride chitosan)-loaded titania nanotubes produced by titanium anodisation.^184^ *In vitro* they demonstrated that these techniques can inhibit *S. aureus* and *S. epidermidis* adhesion. Along with rat model study, they showed a good biocompatibility with osteogenic cells.^185^

#### Other mechanisms

##### Competing interaction molecules

*Heparin.* Generally, *Staphylococcus* adheres to fibronectin using MSCRAMMs on the bacterial surface. Heparin competes with bacterial binding to fibronectin, resulting in a decrease in bacterial adhesion to the extracellular matrix.^186^

## Potential adverse effects from surface modification

Wear particles, from both bearing and implant materials, may have local and systemic implications. Wear particles have been found in liver, spleen and lymph nodes,^187^ and silver nanoparticles have been identified in brain astrocytes.^188^ Wear particles, particularly polyethylene, are one of the primary causes of periprosthetic osteolysis resulting implant loosening and failure. Particle size plays an important role in determining the cellular reaction. Particle sizes >500 nm tend to be engulfed by professional phagocytes using an actin-dependent mechanism,^189^ while small particles are endocytosed by non-professional phagocytic cells. Ti wear particles (1.5–4 µm) have negative effects on osteoblast proliferation and viability,^190^ induce fibroblasts to release matrix metalloproteinases (MMP) resulting in osteolysis^191^ and increase MMP2 and 9 activity, resulting in reduction of bone formation.^192^ Micrometric Ti particles impaired Saos-2 adhesion strength, migration and proliferation.^193^ Furthermore, Ti wear particles stimulate production of inflammatory cytokines, induce lymphocytes to start a type IV immune reaction^194^ and increase vascular endothelial growth factor (VEGF) expression and p44/42 mitogen-activated protein kinase (MAPK) activation in monocytes and macrophages.^195^

Ti dioxide (TiO_2_) is widely used for nanoscale surface modification; however, wear nanoparticles specific to TiO_2_ may have adverse effects. TiO_2_ nanoparticles have been shown to disseminate to heart, lung and liver and can cross the placenta.^196,197^ They have been shown to transfer to offspring and affect the cranial nerve systems in a mouse model,^198^ have multiple immunomodulatory effects and may be associated with genotoxicity.^199^ In the local environment, TiO_2_ nanoparticles have been shown to adversely affect cell migration and MSC differentiation in rats.^200^ However, no long-term clinical studies have shown any adverse effects from the dissemination of TiO_2_ particles.

## Conclusion and future perspective

Prosthetic and bone infections are devastating to patients and healthcare services. We have reviewed the manner in which bacteria interact with implants and host cells and developing surface modification strategies using titanium implants to prevent bacterial adhesion, while maintaining or improving implant osseointegration. Many surface modification strategies have been developed over recent years with some promising success in vitro, but many have yet to find in vivo or clinical use. We would anticipate the adoption of these promising surface modifications to help prevent bacterial colonisation of implants in the future and provide better treatment options.
